# Clinical Manifestations and Outcomes of Budd-Chiari Syndrome in Children: A Single-Centre Study

**DOI:** 10.7759/cureus.48418

**Published:** 2023-11-07

**Authors:** Hassan Bashir, Maryam Mazhar, Iqtadar Seerat, Nazia Iqbal, Mehwish Imtiaz

**Affiliations:** 1 Department of Paediatric Gastroenterology and Hepatology, Pakistan Kidney and Liver Institute, Lahore, PAK

**Keywords:** radiological findings, clinical manifestations, liver, hepatic, bcs, budd-chiari syndrome (bcs)

## Abstract

Introduction

Budd-Chiari syndrome (BCS) is a rare cause of ascites in children, and its clinical manifestation depends upon the extent and rapidity of the occlusion of hepatic veins. This study aimed to identify the clinical manifestations, causes, treatment options, and outcomes of BCS in children.

Materials and methods

A retrospective descriptive study of BCS in children under 15 years of age was conducted. This study was approved by the Pakistan Kidney and Liver Institute and Research Centre on June 23, 2023, with approval number 0128. The patients’ medical records from December 2020 to July 2023 were obtained from Sisoft Healthcare Information System. In this study, we employ a set of predetermined questions to retrieve relevant data retrospectively and then organise it in Excel spreadsheets. SPSS version 26 (Armonk, NY: IBM Corp.) was used to analyse the data. Categorical variables are shown as frequencies (%), while continuous variables are reported as mean±SD.

Results

Of 37 (n) patients diagnosed with BCS, 19 (51.35%) were male and 18 (48.65%) were female. The mean age of presentation was 9.8±4.1 years. Ascites are the predominant clinical manifestation (100%), followed by hepatomegaly (37.8%). A total of 45.9% of patients had deranged liver function tests. Chronic BCS is the predominant mode of presentation. Protein C deficiency was present in nine patients (24.3%), two patients (5.4%) had protein S deficiency and two patients (5.4%) had antithrombin III deficiency. Hepatic veins exhibited the highest incidence of obstruction (73.0%). Liver biopsies were done in 15 (40.54%) patients to determine the staging of fibrosis. Eight patients (21.62%) had undergone radiological interventions, two patients had liver transplants and the rest were treated with medications, including anticoagulants.

Conclusion

BCS can present in acute, subacute or chronic forms. Ascites and hepatomegaly should raise the suspicion of BCS in children. Common radiological findings are non-visualisation of the hepatic veins. BCS has a wide range of aetiologies and treatment options. Protein C deficiency is the most predominant procoagulant disorder. Radiological interventions during the acute and subacute forms of BCS usually have excellent results. Liver transplant remains the definite treatment.

## Introduction

Budd-Chiari syndrome (BCS) is a rare liver disease resulting from hepatic venous outflow tract obstruction in the absence of any cardiac disease, such as constrictive pericarditis or congestive heart failure. It is caused by obstruction of the hepatic veins (HVs) and/or supra-hepatic inferior vena cava (IVC) [1]. Based on aetiology, BCS can be classified into primary or secondary forms. Primary BCS is defined by blockage occurring within the lumen of the veins or venules, commonly caused by thrombus, webs or endophlebitis. Conversely, secondary BCS originates from extra-luminal-factors, including tumours, cysts or abscesses, which either infiltrate the lumen or exert external pressure [2].

BCS has a wide range of aetiologies, clinical manifestations and treatment options. The classic presentation of BCS comprises ascites, hepatomegaly and abdominal pain [3,4]. The clinical presentation can range from asymptomatic to acute, chronic or fulminant [5,6]. Acute BCS usually develops within one month without the formation of venous collaterals. It is characterised by refractory ascites, abdominal pain, hepatomegaly, renal failure and raised serum aspartate or alanine aminotransferase levels [4]. Subacute BCS has an insidious onset which is typically followed by a transition to an asymptomatic state within approximately three months, facilitated by developing venous collaterals. Treatment started during the subacute phase has better outcomes. In chronic BCS, the hallmark is the gradual onset of portal hypertension and venous collaterals, often leading to pronounced abdominal distension due to gross ascites. At the same time, liver function tests are minimally affected or normal [7]. Both acute or chronic forms of BCS can result in centrilobular sinusoidal congestion, hepatic cell necrosis, and, eventually, centrilobular fibrosis [7].

Doppler ultrasonography, CT and MRI, combined with the clinical manifestations, are often applied for diagnosing BCS, although angiography is still the gold standard [8-10]. Therapeutic options include medical management (anticoagulants and diuretics) and radiological interventions, including thrombolysis, percutaneous transluminal angioplasty, direct intra-hepatic portosystemic shunt (DIPS), transjugular intra-hepatic portosystemic shunting (TIPS) and orthotopic liver transplantation [11]. This study presents a comprehensive analysis of BCS detailing the diagnostic and therapeutic evaluation of the patients treated in our centre over a period of two and a half years.

## Materials and methods

Retrospective data analysis was conducted at the Pakistan Kidney and Liver Institute and Research Centre after getting approval from the institutional review board. This study received official approval from the Pakistan Kidney and Liver Institute and Research Centre on June 23, 2023, and it is identified by approval number 0128. All the patients diagnosed with BCS on the basis of imaging, of either gender and age less than 15 years, were included in the study. Patients with any other chronic liver disease or age over 15 years were excluded from the study. In this study, we employ a set of predetermined questions to retrieve relevant data retrospectively and then organise it in Excel spreadsheets. It includes demographics, clinical manifestations, investigations, management plans, course and outcome of the disease. Patients’ medical records of the paediatric gastroenterology and hepatology clinic from December 2020 to July 2023 were obtained from the Sisoft Healthcare Information System.

The workup was conducted in accordance with the routine protocols established by our institute. Baseline investigations and thrombophilia screening, including protein C, protein S and antithrombin III levels, were performed in all patients. Other aetiologies, including myeloproliferative disorders, factor V Leiden mutation, antiphospholipid syndrome and Janus kinase (JAK) mutations, were not requested due to insufficient financial resources. Imagings, including Doppler ultrasonography and computed tomography (CT) scan, were done in all patients to diagnose BCS. Liver biopsy was done only in those patients who had ascites free interval. The Meta-Analysis of Histological Data in Viral Hepatitis (METAVIR) Scoring system was employed to determine the fibrosis stage and extent of activity in liver biopsy specimens. Oesophagogastroduodenoscopy (EGD) was done in patients before starting anticoagulants and antiplatelets. Patients with grade 0 and grade 1 oesophageal varices received aspirin, while those with grade 2 and grade 3 oesophageal varices were prescribed factor Xa inhibitors like rivaroxaban. Diuretics such as furosemide and spironolactone were used to treat the ascites.

Following a comprehensive assessment in a multidisciplinary team meeting (MDT), a selected group of patients was recommended for further medical interventions. Specifically, patients presenting with acute and subacute forms of liver conditions underwent various radiological interventions tailored to their individual needs. These interventions were designed to address the specific requirements of their cases. In cases where patients were experiencing liver failure, liver transplantation was considered a viable treatment option. Decisions regarding radiological interventions and liver transplants were made with a careful evaluation of each patient's medical condition, ensuring that the most suitable and effective treatment approach was chosen to optimise their clinical outcomes. This approach underlines the importance of personalised and multidisciplinary care in managing complex liver conditions.

SPSS version 26 (Armonk, NY: IBM Corp.) was used to analyse the data and develop frequency tables. Categorical variables, such as gender, clinical manifestations and biopsy findings were presented as frequencies and percentages (%), while continuous variables such as age were described as mean±standard deviation (SD).

## Results

A total (n) of 37 patients met the inclusion criterion for the study. Among them, 19 (51.35%) were male and 18 (48.65%) were female. The mean age of presentation was 9.8±4.1 years. The median age of presentation was 11 years, with the earliest presentation at one year. The predominant clinical manifestation was ascites. All patients (100%) had ascites, confirmed by radiological imaging. Only 14 (37.8%) patients had hepatomegaly on physical examination, and classic triad was present in a few patients only (Table 1).

**Table 1:** Frequencies of various clinical manifestations in Budd-Chiari syndrome. The data have been represented as counts (n) and percentages (%).

Clinical features	Count (n)	Percentage (%)
Ascites	37	100%
Hepatomegaly	14	37.8%
Splenomegaly	10	27.0%
Upper GI bleed	9	24.3%
Jaundice	8	21.6%
Abdominal pain	5	13.5%
Classic triad	4	10.8%

Seventeen (45.9%) patients had presented with chronic BCS, followed by acute BCS in 11 (29.7%) patients and subacute BCS in nine (24.3%) patients. Seventeen patients (45.9%) had elevated transaminases. Protein C deficiency was present in nine (24.3%) patients, and two (5.4%) patients had protein S deficiency, of which one (2.7%) patient also had protein C deficiency. Antithrombin III deficiency was present in only two (5.4%) patients. Twenty-five (67.6%) patients have some other aetiology for which they were not tested.

Radiological findings showed hepatic veins (HV) as the predominant site of obstruction. About 27 (73.0%) patients had all three hepatic veins blocked, while eight (21.6%) patients had two hepatic veins blocked, and two (5.4%) patients had only one hepatic vein blocked (Table 2).

**Table 2:** Radiological mapping of venous obstruction sites in Budd-Chiari syndrome. The data have been represented as counts (n) and percentages (%). HV: hepatic veins; IVC: inferior vena cava

Site of obstruction	Count (n)	Percentage (%)
Hepatic veins (HV)	27	73.0%
Inferior vena cava (IVC)	2	5.4%
Both HV and IVC	8	21.6%
Concomitant portal vein thrombosis	6	16.2%

Oesophagogastroduodenoscopy (EGD) was done in 32 patients prior to the start of anticoagulation. A liver biopsy was done in 15 patients, and all 15 had sinusoidal dilatation, a hallmark of BCS. In patients with chronic Budd-Chiari syndrome (BCS), the predominant findings were fibrosis stages F3 and F4, whereas individuals with acute and subacute BCS typically exhibited fibrosis stages F1 and F2 (Table 3).

**Table 3:** Clinical and histopathological findings of the EGD and liver biopsy. The data have been represented as counts (n) and percentages (%). Total number of EGDs=32; total number of liver biopsies=15 EGD: Oesophagogastroduodenoscopy

Procedure	Clinical/histopathological findings		Count (n)	Percentage (%)
Oesophagogastroduodenoscopy (EGD)	Normal oesophageal mucosa		10	31.25%
	Grade 1 oesophageal varices		4	12.5%
	Grade 2 oesophageal varices		6	18.75%
	Grade 3 oesophageal varices		9	28.12%
Liver biopsy	Sinusoidal dilatation		15	100%
	Cirrhosis		5	33.34%
	Necrosis		9	60%
	Fibrosis	F0	0	0%
		F1	2	13.34%
		F2	4	26.66%
		F3	4	26.66%
		F4	5	33.34%
	Activity	A1	7	46.67%
		A2	5	33.34%
		A3	2	13.33%

Antiplatelets and different anticoagulants were started for management in 23 (62.16%) patients. Patients with grade 1 oesophageal varices were advised to take aspirin, while those with grade 2 and grade 3 varices were recommended to receive factor Xa inhibitors like rivaroxaban and apixaban. Aspirin was started in five (13.5%) patients, rivaroxaban in 15 (40.54%) patients, apixaban in three (8.1%) patients, and two (5.4%) patients were started on warfarin. Diuretics, including furosemide and spironolactone, were administered to all patients with ascites. The symptoms showed a significant reduction following the use of the medications.

Patients diagnosed with acute and subacute forms of Budd-Chiari syndrome (BCS) were the primary candidates for radiological interventions. A total of eight patients (21.62%) underwent invasive procedures. Three patients with acute BCS, two with subacute BCS and one with chronic BCS had venoplasty. Radiological interventions such as transjugular intra-hepatic portosystemic shunt (TIPS) and direct intra-hepatic portacaval shunt (DIPS) were performed on one patient each, both of whom presented with subacute forms of BCS. After the interventions, these patients achieved complete symptom resolution and have been undergoing follow-up for one year.

Patients who had liver failure underwent living donor liver transplantation. In this study, two patients (5.4%) had liver transplants; one died after one year due to chronic rejection, while the other remained healthy after 18 months of post-liver transplant.

## Discussion

BCS, although a rare disease in the West, has been frequently described in studies done in Asia, with a prevalence of up to 7.4% in India [12,13]. BCS is frequently reported in adults with chronic liver disease; however, data are scarce in the literature related to BCS in paediatrics. Like the present study, various paediatric studies have described a higher prevalence in males and diverse age distribution at the time of diagnosis [14]. Hepatovenocaval syndrome, a rare vascular disease, is distinguished by the presence of membranous obstruction in the IVC, which is typically caused by a bacterial infection [15]. In the current study, hepatovenocaval syndrome was not observed in any of the patients.

BCS manifests clinically if two or more hepatic veins or supra-hepatic IVC is blocked [1]. Similar to the study in India, the present study shows that hepatic veins are the predominant site of obstruction. However, in this study, two patients with single hepatic vein blockage also had clinical manifestations of BCS. Isolated IVC block is rarely a cause of BCS, and the present study is consistent with that [16].

Acute forms of BCS rapidly develop hepatocellular necrosis since venous collaterals have not formed. Subacute forms of BCS develop collaterals over three months. Chronic BCS develops bridging fibrosis and features of cirrhosis. Liver biopsy findings in the present study are consistent with the literature [5].

According to the literature, the subacute form is the most prevalent form of clinical presentation in BCS [17]. However, in the present study, the majority of patients presented with chronic BCS. This could be due to a lack of understanding and health resources in our part of the developing world.

Detailed aetiological workup was not done in the present study due to insufficient financial resources. However, anticoagulant proteins such as protein C, protein S and antithrombin III activity levels were measured. Corresponding to our findings, a study from the United Kingdom shows that protein C deficiency is frequent among procoagulant disorders [18]. However, this is not very significant as reduced protein C activity can also be attributed to liver dysfunction or recent thrombus formation, and similar effects may be observed in the cases of protein S and antithrombin III.

According to one study from Western India, hereditary thrombophilia is the leading cause of BCS [19]. Eleven patients in the present study were products of consanguineous marriage. However, none of the patients in the present study had a positive family history of thrombophilia.

Ascites and hepatomegaly are the predominant clinical manifestations of the BCS, consistent with the present study [18]. However, abdominal pain was not a common presenting complaint in the present study, and thus, the classic triad, defined as abdominal pain, ascites and hepatomegaly, was less common, unlike other studies [20].

The present study supports the literature that patients with BCS can present with normal or mild to moderately elevated transaminases and deranged bilirubin levels. A few patients also had deranged prothrombin time (PT) and International Normalised Ratio (INR) [18].

According to multicentre European cohort BCS, concomitant portal vein thrombosis was present in 18% of the patients. The present study is consistent with that, as 16.2% of patients in the present study also had concomitant portal vein thrombosis [21].

A recent study provides insight into the step-wise management of BCS. Several factors, including acuity of presentation, the site of the blockage, the liver’s structural anatomy and local expertise, influence the management of BCS. Lifelong anticoagulants and antiplatelets can be prescribed initially. Literature shows that direct oral anticoagulants (DOACs) are safe in the medical management of BCS [11]. In the present study, most patients were prescribed direct oral anticoagulants such as rivaroxaban and apixaban, while few were given antiplatelets such as aspirin.

In acute and subacute forms of presentation or after the failure of medical treatment, radiological interventions such as stenting/angioplasty, TIPS or DIPS can be performed [16]. In the present study, successful venoplasty and TIPS/DIPS were done in patients who presented with acute and subacute forms of BCS. Successful venoplasty was also done in one patient with chronic BCS.

There are reported cases of successful liver transplants in paediatric patients. Patients with BCS who receive liver transplants have a five-year survival rate of up to 95% [22]. In the present study, two patients had liver transplants; one died after one year due to chronic rejection, while the other remained healthy after 18 months of post-liver transplant.

While this study provides valuable insights into understanding the clinical approach to BCS, it is essential to acknowledge a few limitations. These limitations encompass the absence of a comprehensive aetiological workup aimed at identifying the underlying causes of BCS. This deficiency stems from limited financial sources available for conducting such investigations. Additionally, delayed presentations to our clinic, driven by a lack of health awareness, resulted in a substantial portion being categorised as chronic BCS cases. The capacity to carry out liver biopsies was constrained to a limited number of patients due to the ongoing presence of ascites in a significant proportion of cases.

## Conclusions

Budd-Chiari syndrome, although a rare disease, has diverse clinical presentation. BCS can present in acute, subacute or chronic forms. Ascites and hepatomegaly should raise the suspicion of BCS in children. Radiological findings include non-visualisation of the hepatic veins or supra-hepatic inferior vena cava on Doppler ultrasonography and CT scan.

BCS has a wide range of aetiologies and treatment options. In this study, the aetiology of BCS is predominantly idiopathic, with protein C deficiency emerging as the primary procoagulant disorder. A liver biopsy is important for definitive diagnosis. Esophagogastroduodenoscopy must be done prior to the start of anticoagulants or antiplatelets to rule out any varices. Diuretics are the mainstay for treating ascites. Radiological interventions during the acute and subacute forms of BCS usually have excellent results, especially in slightly older children. A liver transplant remains the definitive treatment of BCS.
